# Influence of game features on attention in adults

**DOI:** 10.3389/fpsyg.2023.1123306

**Published:** 2023-05-09

**Authors:** Courtney L. Gallen, Jessica N. Schachtner, Roger Anguera-Singla, Joaquin A. Anguera, Adam Gazzaley

**Affiliations:** ^1^Department of Neurology, University of California, San Francisco, San Francisco, CA, United States; ^2^Neuroscape Center, University of California, San Francisco, San Francisco, CA, United States; ^3^Department of Psychology, University of Arizona, Tucson, AZ, United States; ^4^Department of Psychiatry, University of California, San Francisco, San Francisco, CA, United States; ^5^Department of Physiology, University of California, San Francisco, San Francisco, CA, United States

**Keywords:** attention, ADHD symptoms, game features, continuous performance task, reward responsiveness

## Abstract

**Introduction:**

The incorporation of game features into cognitive tasks can inform us about the influence of reward and motivation on attention. Continuous performance tasks (CPTs), designed to assess attention abilities, are examples of cognitive tasks that have been targeted for the addition of game features. However, previous results have been mixed regarding how game elements affect attention abilities and task performance.

**Methods:**

Here, we studied if there were factors that predict which individuals exhibit changes in attention from game features added to a CPT. Participants (*N* = 94, aged 21–71) played a traditional CPT and a game CPT with identical mechanics, but featured engaging game elements (aesthetics, storyline, competition, feedback, and reward).

**Results:**

We first found corroborating evidence that game features have mixed effects on attention performance: most attention metrics of interest exhibited no overall difference between the traditional and game CPT, while game elements reduced performance for a few metrics. Importantly, we also found that specific behavioral and demographic profiles predicted individual differences in performance on the game CPT compared to the traditional CPT. Those with more attention difficulties (ADHD symptoms), more reward responsiveness, and younger adults performed better on the game CPT while, conversely, those with fewer ADHD symptoms, less reward responsiveness, and older adults performed better on the traditional CPT.

**Discussion:**

These findings provide insights into how game features can influence attention in different individuals and have important implications for the use of game elements in cognitive tasks and training interventions.

## Introduction

Reward and motivation can strongly influence attention and other aspects of cognition. There is rapidly growing interest in adding game features to computerized cognitive tasks (e.g., reward, feedback, storylines, and competition) to provide unique insights into how motivational processes affect performance. In addition, game elements may improve data quality for cognitive assessments and provide deeper and longer-term engagement in cognitive interventions (Lumsden et al., 2016; Vermeir et al., 2020; Khaleghi et al., 2021).

Continuous performance tasks (CPTs) that assess attention (Esterman and Rothlein, 2019) have been a frequent target for the addition of game features, where participants maintain their focus over time to respond to target stimuli and inhibit responses to non-target stimuli. Attention is a key component of cognitive control abilities (Mishra and Gazzaley, 2014; Anguera and Gazzaley, 2015) and has impact on real-world functioning [e.g., academic success (Arffa, 2007; Best et al., 2011; Gallen et al., 2023)] and physical and mental health (Moffitt et al., 2011; Hinshaw and Scheffler, 2014). There is thus a need to understand how to maximize an individual’s attention, potentially with game elements. However, previous work has yielded mixed results (Lumsden et al., 2016; Khaleghi et al., 2021), with some showing game features improve attention (Delisle and Braun, 2011; Shaw et al., 2016) and others finding they impair it (McPherson and Burns, 2007, 2008; Miranda and Palmer, 2014). Although these mixed findings may be due to small samples and heterogenous designs (Khaleghi et al., 2021), the contribution of meaningful individual differences to these effects has not been well studied.

To shed light on this unresolved issue, we examined the role of individual differences in predicting changes in attention from added game features, focusing on self-reported demographic and behavioral characteristics. Our three primary variables of interest (i.e., those that we had strongest hypotheses for prediction) were: (1) ADHD symptoms, (2) reward responsiveness, and (3) age. First, although individuals with ADHD often have poorer performance on traditional CPTs, there is evidence that their attention deficits are context dependent. Those with ADHD often have equivalent, or even superior, performance on more engaging tasks that also demand prolonged attentional control [e.g., videogames and computer games; (Hinshaw, 2017)]. Second, performance difficulties on traditional CPTs may also be related to motivation and arousal. Indeed, some theories of ADHD dysfunction point to insufficient intrinsic motivation and reward responsiveness (Volkow et al., 2009; Beauchaine and McNulty, 2013). Individuals with ADHD often require higher levels of immediate rewards and therefore find traditional CPTs to be boring and tedious (Hinshaw, 2017), resulting in low motivation and poor performance (Castellanos et al., 2006). In contrast, tasks with game features may be more stimulating, thus motivating engagement and enhancing performance. Third, aging has pronounced effects on both attention (Zanto and Gazzaley, 2019; Vallesi et al., 2021) and reward processing (Green et al., 1993). Moreover, older adults may have different preferences for cognitive paradigms compared to young adults, as they tend to rate interventions with video game elements as less enjoyable (Boot et al., 2013). Thus, there is strong prior evidence that game features may alter attention abilities based on an individual’s profile, where those with: (1) more ADHD symptoms, (2) more reward responsiveness, and (3) younger individuals may exhibit enhanced performance with added game elements. Conversely, those with lower ADHD symptoms, lower reward responsiveness, and older individuals may exhibit impaired performance due to distraction induced by such features.

In addition, we examined potential effects of three secondary variables that might also influence attention changes from added game features: (1) frequency of video game play, (2) gender, and (3) education. Individuals who play video games more often have greater exposure to various game elements, which may affect their responsivity to game features added to a cognitive task. For example, prior work has shown that video game players have stronger attention abilities (Green and Bavelier, 2003; Dye et al., 2009; Bavelier and Green, 2019). These individuals may be better able to sustain their attention during a task with added game elements because of their familiarity and practice in such contexts. Additionally, other basic sociodemographic characteristics such as gender and education are important to consider as they may impact frequency of video game play (Lucas and Sherry, 2004; Ogletree and Drake, 2007) as well as broader attention abilities (Alley et al., 2007; Jefferson et al., 2011; Lövdén et al., 2020). Therefore, we finally sought to examine whether video game play, gender, and education influence the effects of game features on attention.

To examine if these factors predict how game elements influence attention, participants performed a traditional CPT and a game CPT that had identical task mechanics, but the latter included art, music, reward, feedback, storyline, and competition. We then asked how individual differences moderated changes in performance between the traditional and game CPT to identify factors that predicted changes in attention from game elements. Specifically, we investigated game-based influences on three performance metrics that are commonly examined in the CPT literature to index various aspects of attentional control: (1) response times (RTs) for target stimuli were used as a measure of attention processing speed (Anguera et al., 2013), (2) variability of response times (RTV) was used as a measure of attention consistency [i.e., the consistency of RT-based attentional deployment across target stimuli (MacDonald et al., 2006; Esterman et al., 2012; Ziegler et al., 2019; Kollins et al., 2020)], and (3) d-prime (comparing correct target responses and incorrect non-target responses) was used as a measure of performance accuracy (Esterman et al., 2012; Esterman and Rothlein, 2019). In doing so, we aim to better understand how and why game elements shape aspects of attention across individuals.

## Materials and Methods

### Participants and study procedures

**Figure 1:**
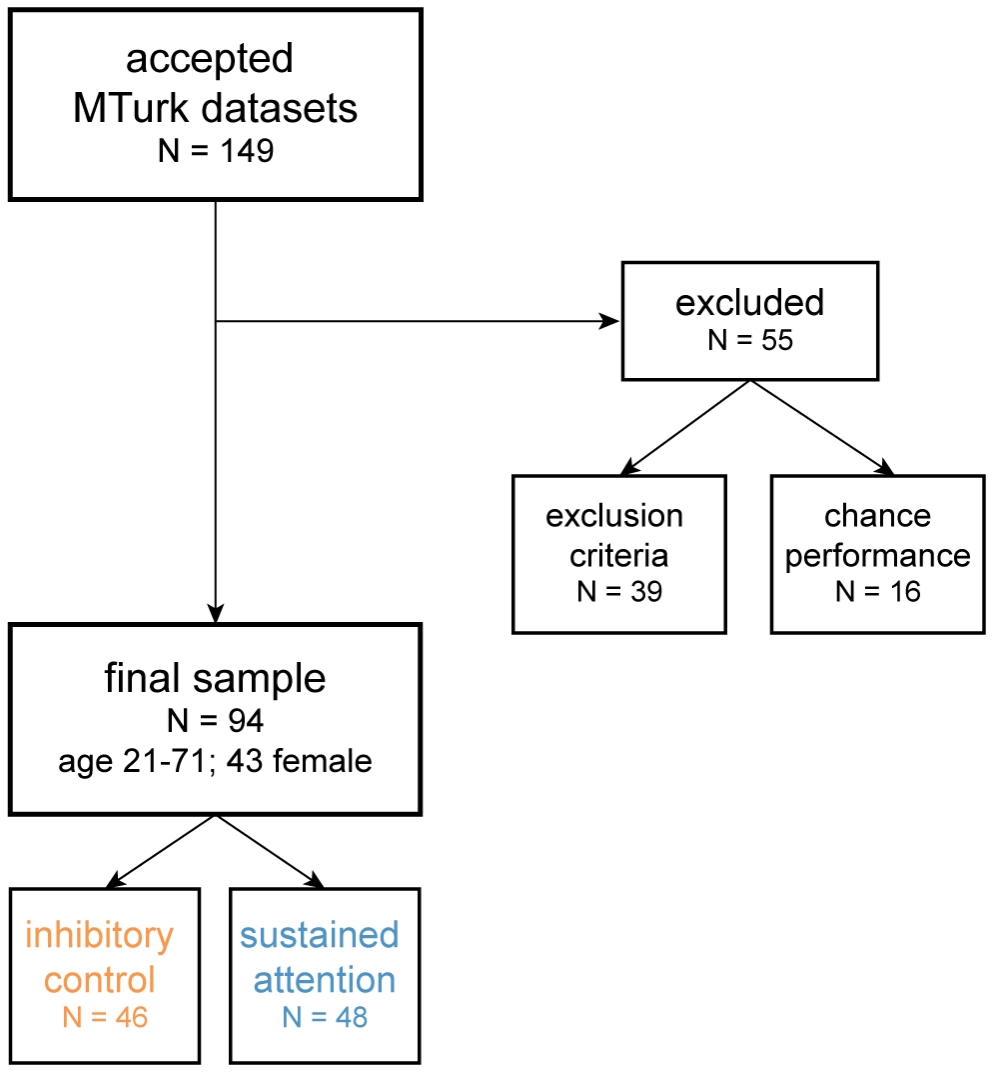
CONSORT flow diagram. We collected data from 149 adults (age 18 or older) through Amazon Mechanical Turk (MTurk). We first excluded 55 participants from all analyses because they did not meet our inclusion criteria (e.g., incomplete datasets, self-reported psychiatric or neurological disorders; *N* = 39) or had chance or below chance performance on either the traditional or game CPT (defined as d-prime ≤0; *N* = 16). The final dataset for analysis included 94 adults aged 21–71 years old (*N* = 43 female), where 46 played the inhibitory control CPT condition (frequent target stimuli; orange) and 48 played the sustained attention CPT condition (infrequent target stimuli; blue).

We recruited 149 adults through Amazon Mechanical Turk (MTurk) who were over the age of 18 for this study (Figure 1). To be eligible for participation, individuals were required to have an MTurk approval rate (marker of work quality) above 95 and have over 50 previous MTurk studies completed and approved, participation criteria that are in accordance with (or more stringent than) previous MTurk studies (Hauser and Schwarz, 2016; Kambanis et al., 2021). To be included in our final analyses, participants were required (1) to have complete datasets and not have experienced any technical difficulties and (2) be native English speakers with normal or corrected-to-normal vision and hearing, living in the United States, and without any self-reported neuropsychiatric and neurological disorders (except for a self-reported ADHD diagnosis). Prior to participation, individuals gave their informed consent in accordance with the Institutional Review Board of the University of California, San Francisco (UCSF) and received $3 as compensation for approximately 30 min of their time. All methods were carried out under the relevant guidelines and regulations for experimental protocols in UCSF IRB #13–10,917.

After collecting datasets from 149 participants, 55 participants were excluded from further data analysis. Thirty-nine participants were excluded for not meeting the inclusion criteria described above: 28 due to incomplete datasets or technical issues, 11 due to self-reported neuropsychiatric or neurological disorders (except ADHD). Another 16 participants were excluded for chance or less than chance performance on the CPT (defined as d-prime ≤0, see Statistical analysis for more details). Exclusion on performance was relatively matched for the traditional and game CPTs (inhibitory control condition: 6 participants on the traditional CPT, 6 participants on the game CPT, and 1 participant on both CPT types; sustained attention condition: 1 participant on the traditional CPT and 2 participants on the game CPT). Our final sample for analysis therefore included 94 participants aged 21–71 years old (Figure 1).

This study was administered through MTurk and Qualtrics. Participants found the study through MTurk, which provided a Qualtrics link where they completed surveys and two CPTs (traditional and game). After the surveys, participants were assigned to one of two CPT conditions (Figure 1) – an inhibitory control condition (N = 46) where target stimuli appear frequently (77% of trials) or a sustained attention condition (N = 48) where targets appear infrequently (22% of trials; see Continuous performance tasks (CPTs) descriptions for more details). The target sample in each condition group was approximately 42, as prior work examining overall effects of game features on task performance or effects of individual differences (i.e., ADHD symptoms) on game feature responsiveness had an average sample size of 41.8 individuals [range: 16–70; (McPherson and Burns, 2007, 2008; Delisle and Braun, 2011; Miranda and Palmer, 2014; Shaw et al., 2016)]. With our final sample sizes for each CPT condition, we were powered to detect correlation-based effects of *r* = 0.4, which corresponds to an approximately 80% (1−β) power estimate at a 95% (1−α) two-tailed significance level.

The two groups were matched on age [*t*(92) = 0.17, *p* = 0.86], gender [X^2^ (1, *N* = 94) = 0.66, *p* = 0.42], handedness [X^2^ (2, *N* = 94) = 1.29, *p* = 0.53], years of education [*t*(92) = −1.27, *p* = 0.21], hours spent playing video games per week [*t*(92) = 1.46, *p* = 0.15], and ADHD symptoms [t(92) = −1.11, *p* = 0.27]. Participants in the inhibitory control condition group had lower reward responsiveness (BAS reward responsiveness subscale) compared to those in the sustained attention condition group [*t*(92) = −2.10, *p* = 0.04]. No participants in our final dataset had a self-reported ADHD diagnosis. See Table 1 for participant demographics in each group.

**Table 1:** Participant demographics for the inhibitory control and sustained attention CPT condition groups.

	Inhibitory control *N* = 46	Sustained attention *N* = 48
Age	40.93 (12.20)	40.50 (12.22)
Gender (F/M)^a^	23/23	20/28
Handedness (R/L/A)^b^	41/4/1	45/3/0
Years of education	14.72 (2.02)	15.25 (2.06)
Video game play (hours per week)	9.91 (8.42)	7.15 (9.87)
ADHD symptoms	6.35 (5.29)	7.52 (4.96)
BAS reward responsiveness	7.96 (2.54)	9.13 (2.84)

Data are presented as mean (standard deviation) unless otherwise noted.

a Number of participants who were female (F) or male (M).

b Number of participants who were right-handed (R), left-handed (L), or ambidextrous (A).

### Survey-based measures

First, participants completed a set of questionnaires assessing basic information: age, gender, handedness, years of education, and how many hours they spent playing video games per week. Next, to assess ADHD symptoms, participants completed the Adult ADHD Self-Report Scale (ASRS-v1.1) Symptom Checklist (Kessler et al., 2007). The questions are based on DSM criteria and assess aspects of inattention and hyperactivity/impulsivity. Participants rate their responses for each question on a Likert scale from 0 (“never”) to 4 (“very often”). To quantify ADHD symptoms, the scores for the first 6 questions were summed, with a maximum of a total score of 24. Higher scores represent more ADHD symptoms. We focused on the first 6 questions from the ASRS as they have been shown to be more sensitive, specific, and accurate in predicting DSM-based clinical ratings of ADHD compared to the full 18 question set (Kessler et al., 2005). As such, it has been recommended that the 6-question “screener” be used for self-reporting ADHD symptoms. To assess motivational systems that underlie approach behaviors that facilitate goal-directed actions, participants completed the Behavioral Inhibition Scale/Behavioral Activation System (BIS/BAS) survey (Carver and White, 1994). We focused on the BAS reward responsiveness subscale, which has been used as a measure of overall reward sensitivity, defined as the ability to experience pleasure in the anticipation or presence of rewarding stimuli (Scheres and Sanfey, 2006; Taubitz et al., 2015). Moreover, prior work has indicated that the reward responsiveness subscale may be a more “pure” measure of BAS than the drive or fun seeking BAS subscales (Taubitz et al., 2015). Participants rate their responses on a Likert scale of 1 (“very true for me”) to 4 (“very false for me”). To quantify BAS reward responsiveness, the scores from the 5 questions for this subscale were summed, with a maximum reward responsiveness score of 20. Higher scores represent more reward responsiveness. If participants had more than one complete survey submission (e.g., answered the survey questions twice), we only analyzed survey data from their first submission.

### Traditional and game continuous performance tasks

After the surveys, participants completed visual continuous performance tasks (CPTs) similar to the Test of Variables of Attention [TOVA; (Greenberg and Waldmant, 1993)], which has been used extensively in our previous work. Of note, TOVA is FDA cleared for the objective assessment of attention deficits and the evaluation of intervention effectiveness in ADHD (Kollins et al., 2020). In this task, participants were instructed to respond using the spacebar key to visual stimuli appearing at the top edge of the computer screen (target stimuli) and to withhold responses to those appearing at the bottom edge of the screen (non-target stimuli). In the traditional version of the CPT based on TOVA, no feedback or rewards are given.

Participants were assigned to one of two groups that differed based on their CPT condition (i.e., an inhibitory control or sustained attention condition, which varied in the number of target stimuli). Both CPT conditions contained 324 trials that were split into 2 task blocks and lasted approximately 10 min in total. The inhibitory control condition (frequent targets) contained 126 targets and 36 non-targets per block, yielding 252 total targets. The sustained attention condition (infrequent targets) contained 36 targets and 126 non-targets per block, yielding 72 total targets. Stimuli were presented for 100 ms in 2000 ms intervals. We note that, unlike the traditional version of TOVA where participants complete both the inhibitory control and sustained attention conditions, we limited participants to only one task condition in this study. This methodological decision was due to time commitments typical to MTurk studies and given that each participant played both a traditional and game CPT.

The CPTs were programmed in Unity™ and the task was completed on the participants’ personal computers. Once they were assigned to a CPT condition group (inhibitory control or sustained attention), participants played both a traditional version of the CPT and a game version of the CPT. The CPT type (traditional or game) was counterbalanced across participants.

**Figure 2:**
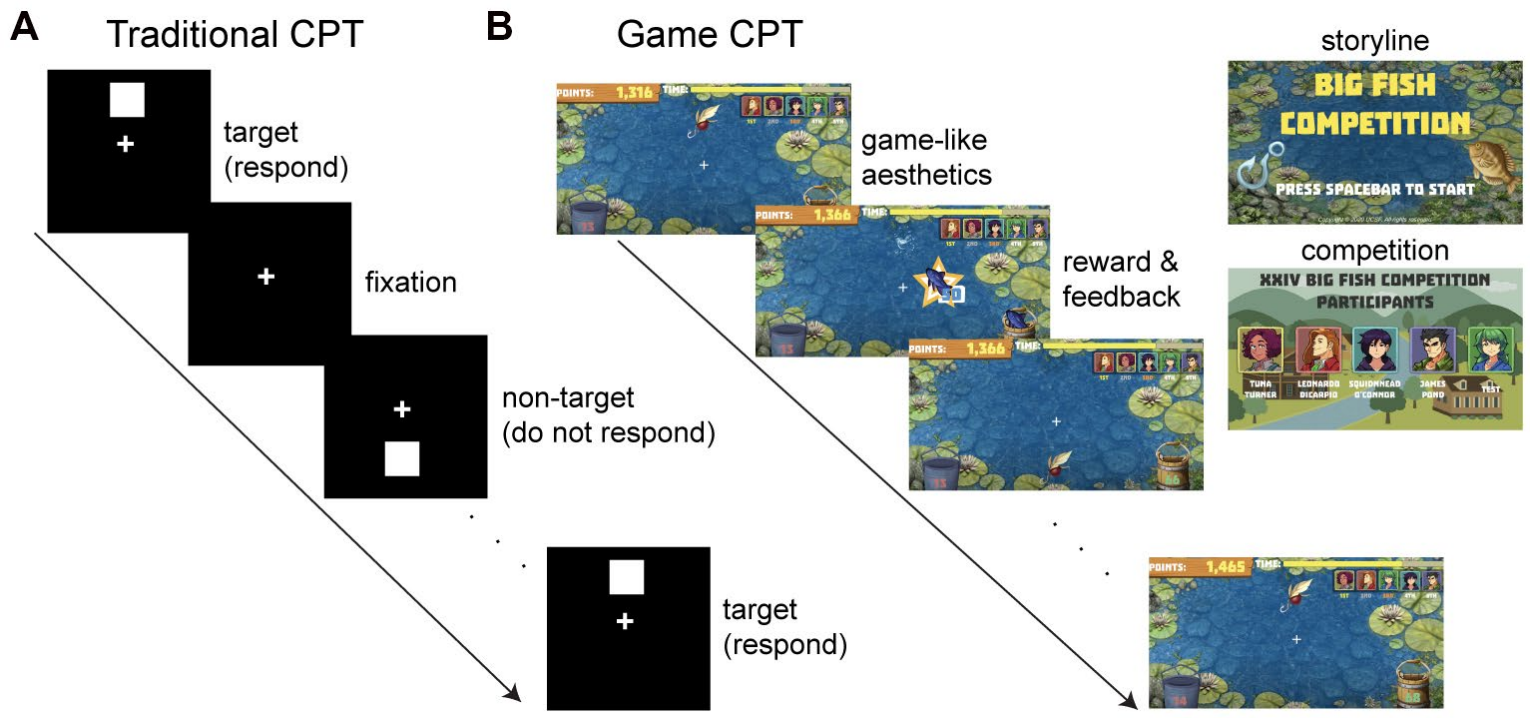
Traditional and game CPTs. **(A)** In the traditional CPT, participants were instructed to respond to white squares appearing at the top of the screen (targets) and withhold responses to those appearing at the bottom of the screen (non-targets), while maintaining fixation on a white cross in the center of the screen. **(B)** In the game CPT, participants instead responded to fishing lures at the top of the screen (targets) and withheld responses to lures at the bottom of the screen (non-targets). The mechanics of the game CPT were identical to the traditional CPT; however, we added several game elements, such as game aesthetics (background music and visuals), reward and feedback (catching fish and associated points), as well as storyline and competition (participants were participating in a fishing competition).

*Traditional CPT*. The traditional CPT was modeled after TOVA. Participants were instructed to respond using the spacebar when a white square appeared at the top of the screen (targets) and to withhold responses to white squares that appeared at the bottom of the screen (non-targets; Figure 2A).

*Game CPT*. The game CPT was identical to the traditional CPT in terms of mechanics (e.g., stimulus presentation time, inter-trial interval, number of target and non-target stimuli), but incorporated added game elements (Figure 2B). First, the game CPT included a storyline and competition, where participants were participating in a fishing competition with the goal of catching the “most-desirable” fish by responding as quickly and accurately as possible. Participants “competed” against the scores of preset avatars and competed against themselves by trying to reach a higher score during the second task block, compared to the first task block.

Second, instead of simple white squares as the stimuli, fishing lures were used to advance the storyline. Participants were instructed to respond using the spacebar when a fishing lure appeared at the top of the screen (targets) and to withhold responses to fishing lures that appeared at the bottom of the screen (non-targets). Other game aesthetics included water noises in the background, engaging water visuals, and background music.

Third, audiovisual reward and feedback were implemented, where participants received 1 of 8 possible fish as a reward if they responded correctly to targets and received 1 of 3 pieces of “junk” (e.g., driftwood) if they incorrectly responded to non-targets. Participants were further incentivized to respond quickly to target stimuli with a point system based on their own response times (RTs) calculated from early task trials, where faster RTs were rewarded with more desirable fish, worth a larger number of points. Specifically, point thresholds for the fastest and slowest RTs were calculated based on the median and median absolute deviation (MAD) from the participant’s first 5–10 correct RTs (at least 5 correct RTs were required for calculations, and up to 10 correct RTs were used). All responses to non-targets and responses with RTs < 150 ms were considered incorrect and not included in these calculations. Subsequent RTs that were faster than 2 MADs below the median RT were rewarded with the fish worth the most points (500 points) and those that were slower than 2 MADs above the median RT were rewarded with the fish worth the least points (1 point). Points for the remaining 6 fish rewards were linearly interpolated within these participant-specific RT bounds. Prior to calculating these thresholds, fish and their associated points were randomly presented for early correct responses. Finally, if participants incorrectly responded to non-target stimuli, they lost a point for that trial and received an associated piece of “junk.”

### Statistical analysis

CPT data were processed to classify trials as correct responses to target stimuli (“hits”) and incorrect responses to non-target stimuli (“false alarms”). In-line with standard TOVA task processing, all trials with response times faster than 150 ms were discarded and not included our analyses. To quantify task performance, three metrics commonly used in the CPT literature were examined. Response time (RT; mean across correct target “hit” trials) was used as a measure of processing speed. Response time variability (RTV; standard deviation of RTs across correct target “hit” trials) was used a measure of attention consistency. D-prime was used measure of performance accuracy across trials, by comparing the proportion of hits and false alarms (*Z*_hit_ − *Z*_falsealarm_). Calculations of d-prime revealed that 16 participants (*N* = 13 in the inhibitory control condition and *N* = 3 in the sustained attention condition) had chance or below chance performance (d-prime scores ≤0 on either the traditional or game CPTs), indicating that they were not playing the task appropriately or did not understand the instructions. These 16 participants were excluded from all final analyses (Figure 1).

We first compared performance on the traditional and game CPT across all individuals. To examine whether there was a relationship between traditional CPT and game CPT performance, Spearman correlations were calculated between traditional and game performance metrics (e.g., a correlation between traditional RT and game RT), separately for each CPT condition. To examine differences in performance between the traditional and game CPTs, we conducted repeated-measures ANOVAs with a within-subjects factor of CPT type (traditional or game) and a between-subjects factor of CPT condition (inhibitory control or sustained attention), separately for each of the performance metrics. We then interrogated changes between traditional and game CPT performance using paired-samples t-tests on each of our performance metrics, separately for each CPT condition.

Next, we examined how individual differences moderated the effect of game features on CPT performance. We generated a “game change” score by calculating the difference in performance on the two types of CPTs. Values above zero represent a game CPT performance advantage (or traditional CPT disadvantage), while values below zero represent a traditional CPT advantage (or game CPT disadvantage). Then, potential moderators of the game change score were examined, by testing whether individual difference metrics of interest could predict game change scores. To do so, we conducted Spearman correlations between the moderator and performance change score (e.g., correlation between ADHD symptoms and game change score for RT).

In all correlation-based analyses, we conducted non-parametric Spearman correlations (denoted as r_s_) to reduce influence from potential extreme values. This was intended to use as much available data as possible in our correlation analyses, without imposing additional outlier exclusion criteria that would further limit our sample sizes (although note we did exclude some participants based on the d-prime threshold discussed above). When we compared the magnitude of correlations (e.g., to assess whether a relationship was stronger for the traditional or game CPT), we converted Spearman’s correlation coefficients to Pearson correlation coefficients using the formula described by Myers and Sirois (2006). We then compared the magnitude of the resulting correlation coefficients using the formula described by Cohen (Cohen et al., 2003). Data were analyzed using custom code written in Python that used a number of libraries, including numpy, scipy, pandas, and pingouin. Data were visualized in Python using the seaborn and matplotlib libraries. We set a significance threshold of *p* ≤ 0.05 and report non-significant “trends” at *p* ≤ 0.10.

## Results

### Comparisons between traditional and game CPT performance

**Figure 3:**
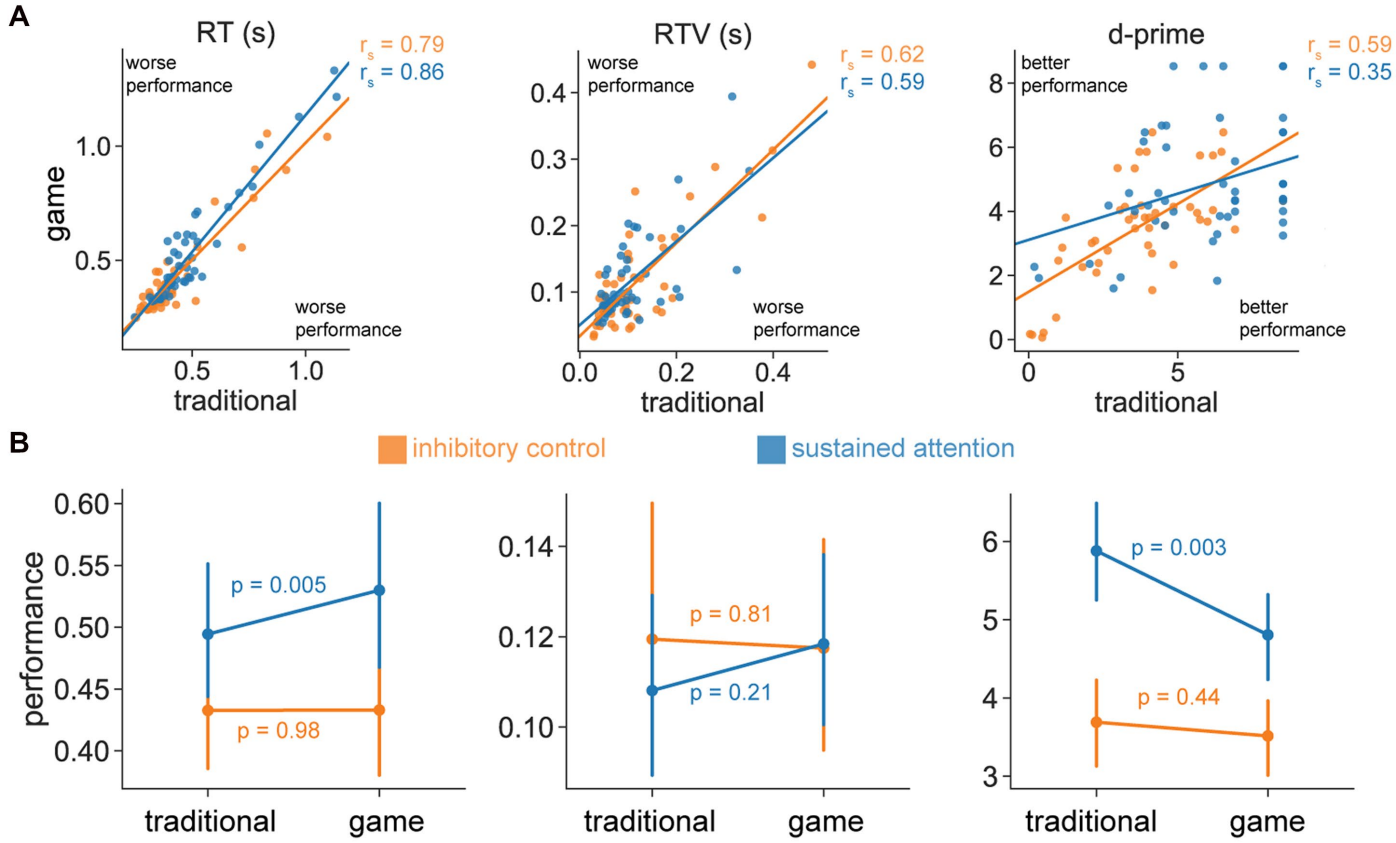
Effects of game features on CPT performance. **(A)** Relationship between traditional and game CPT performance for RT (left), RTV (middle), and d-prime (right) for the inhibitory control and sustained attention conditions (orange and blue, respectively). Note that statistical values represent Spearman correlations (r_s_), while the line represents the linear fit between the two variables (for visual purposes only). **(B)** Differences in performance between traditional and game CPT performance for RT (left), RTV (middle), and d-prime (right) for the inhibitory control and sustained attention conditions (orange and blue, respectively). Points represent the mean and error bars represent 95% bootstrapped confidence intervals. *p*-values reflect uncorrected values.

We first quantified the relationship between performance on the traditional and game CPT, for our performance metrics of interest (RT, RTV, and d-prime). For the inhibitory control condition, we found strong correlations between the traditional and game CPT for all performance metrics [RT: r_s_(44) = 0.79; RTV: r_s_(44) = 0.62; d-prime: r_s_(44) = 0.59; all *p* < 0.001; Figure 3A]. We found similar relationships for the sustained attention condition [RT: r_s_(46) = 0.86, *p* < 0.001; RTV: r_s_(46) = 0.59, *p* < 0.001; d-prime: r_s_(46) = 0.35, *p* = 0.02; Figure 3A]. The pattern of these results suggests that individuals who performed well on the traditional CPT also performed well on the game CPT.

To examine differences in performance between the traditional and game CPTs, we conducted repeated-measures ANOVAs with a within-subjects factor of CPT type (traditional or game) and a between-subjects factor of CPT condition (inhibitory control or sustained attention). For RT, we observed a main effect of CPT type [*F*(1,92) = 4.77, *p* = 0.03], a “trend” toward a main effect of CPT condition [*F*(1,92) = 3.69, *p* = 0.06], where participants were faster for the inhibitory control condition and the traditional CPT. We also observed a significant interaction between type and condition [*F*(1,92) = 4.62, *p* = 0.03], suggesting that performance differences due to game features depended on the CPT condition. Directly comparing performances between the traditional and game CPTs for each condition (Figure 3B, left), we found that participants were faster for the traditional CPT only for the sustained attention condition [*t*(47) = −2.93, *p* = 0.005], but did not exhibit significant differences in performance based on CPT type for the inhibitory control condition [*t*(45) = −0.03, *p* = 0.98]. Note that the difference between CPT types for the sustained attention condition would pass a correction for testing the three performance metrics (*p*_corrected_ = 0.02).

For RTV, we did not observe any significant main effects for CPT type [*F*(1,92) = 0.53, *p* = 0.47] or CPT condition [*F*(1,92) = 0.11, *p* = 0.74], or an interaction between type and condition [*F*(1,92) = 1.16, *p* = 0.28]. Directly comparing performances between the traditional and game CPTs for each condition (Figure 3B, middle), we similarly found that performance was not significantly different based on CPT type for either the inhibitory control [*t*(45) = 0.24, *p* = 0.81] or sustained attention [*t*(47) = −1.28, *p* = 0.21] conditions.

For d-prime, we observed main effects of CPT type [*F*(1,92) = 9.00, *p* = 0.003] and CPT condition [*F*(1,92) = 24.95, *p* < 0.001], where participants were more accurate for the sustained attention condition and the traditional CPT. We also observed a significant interaction between type and condition [*F*(1,92) = 4.65, *p* = 0.03], suggesting that performance differences due to game features depended on CPT condition. Directly comparing performances between the traditional and game CPTs for each condition (Figure 3B, right), we found that participants were more accurate for the traditional CPT only for the sustained attention condition [*t*(47) = 3.10, *p* = 0.003], but did not exhibit significant differences in performance based on CPT type for the inhibitory control condition [*t*(45) = 0.78, *p* = 0.44]. Note that the difference between CPT types for the sustained attention condition would pass a correction for testing the three performance metrics (*p*_corrected_ = 0.01).

Overall, the pattern of these results indicated that performance was sometimes worse on the game CPT compared to the traditional CPT. This effect was observed only for the sustained attention condition in terms of speed (RT) and accuracy (d-prime). All performance metrics for the inhibitory control condition and RTV for the sustained attention condition did not exhibit significant differences between the traditional and game CPTs.

### Individual differences that predict performance changes on the game CPT

Given that the overall findings of whether game features alter attention performance were mixed (i.e., most metrics showed no change, while only RT and d-prime for the sustained attention condition were worse with added game features), we next identified moderators of the game change score: factors that predicted whether individuals performed better (or worse) on the game CPT compared to traditional CPT. We specifically examined 6 factors from participant self-report data: (1) ADHD symptoms, (2) reward responsiveness, (3) age, (4) frequency of video game play, (5) gender, and (6) education. We therefore provide corrected *p*-values as “*p*_corrected_” (corrected for 6 tests) for the initial analyses and then provide only uncorrected p-values for follow-up analyses that interrogate these findings further.

### ADHD symptoms

**Figure 4:**
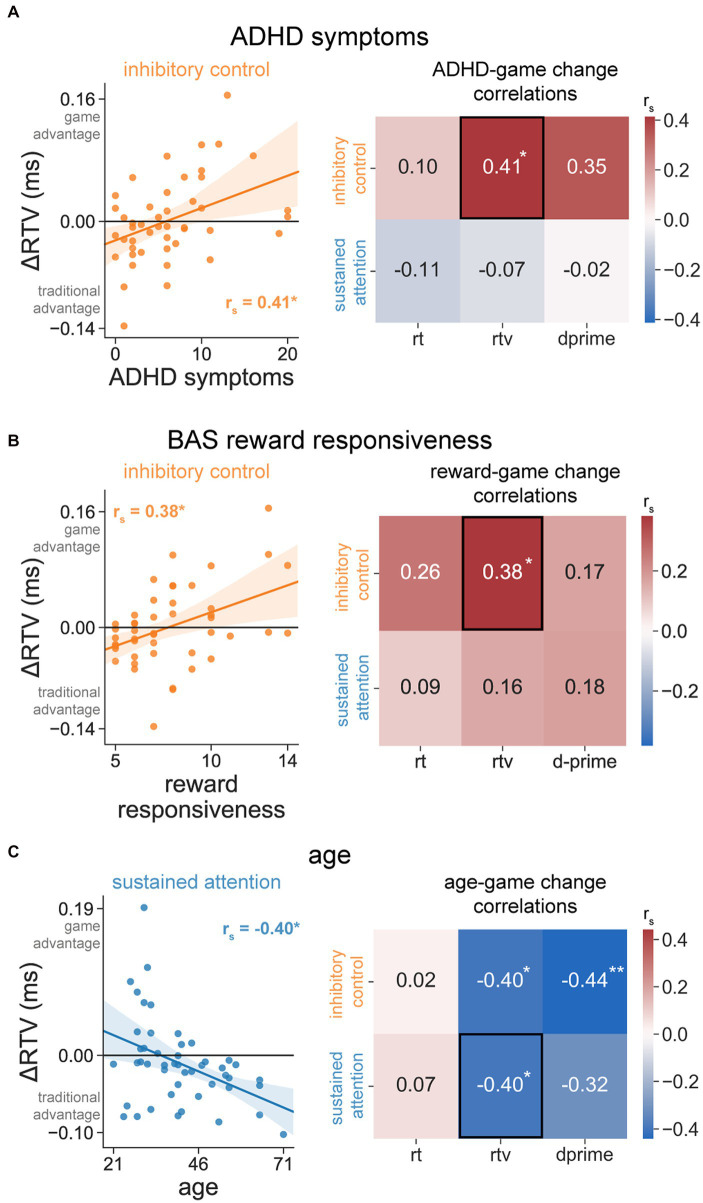
Effects of individual differences on the game CPT performance change. **(A)** ADHD symptoms. Relationship between ADHD symptoms and the game change score for RTV in the inhibitory control condition (left). Spearman correlations between ADHD symptoms and all performance metrics for the inhibitory control (orange) and sustained attention (blue) conditions (right). **(B)** BAS reward responsiveness. Relationship between reward responsiveness and the game change score for RTV in the inhibitory control condition (left). Spearman correlations between reward responsiveness and all performance metrics for the inhibitory control (orange) and sustained attention (blue) conditions (right). **(C)** Age. Relationship between age and the game change score for RTV in the sustained attention condition (left). Spearman correlations between age and all performance metrics for the inhibitory control (orange) and sustained attention (blue) conditions (right). In the scatter plots (left), statistical values represent Spearman correlations (r_s_), while the line represents the linear fit between the two variables (for visual purposes only). Shaded areas in the scatter plots represent 95% bootstrapped confidence intervals. Black boxes around elements in the correlation matrices (right) denote that its representative scatter plot is shown to the left. *p_corrected_ ≤ 0.05; **p_corrected_ ≤ 0.01.

For the inhibitory control condition, ADHD symptoms were positively related to the game change score for RTV [r_s_(44) = 0.41, *p* = 0.004, *p*_corrected_ = 0.02; Figure 4A, left], but not for RT [r_s_(44) = 0.10, *p* = 0.49, *p*_corrected_ = 1.0] or d-prime [r_s_(44) = 0.35, *p* = 0.02, *p*_corrected_ = 0.12; Figure 4A, right]. The direction of these results suggests that the greater the ADHD symptoms, the greater the advantage for the game CPT compared to the traditional CPT, specifically in terms of attention consistency (RTV). Importantly, this metric (inhibitory control RTV) showed no mean difference between the traditional and game CPTs (Figure 3B, middle), suggesting that ADHD symptoms index variability in performance changes (increases or decreases) with added game features in the absence of overall effects.

Given that we found a relationship between ADHD symptoms and the game change score for RTV in the inhibitory control condition, we next examined the relationships between ADHD symptoms and this metric separately for each CPT type (game and traditional), to test whether ADHD symptoms predicted the game change score because it correlated with traditional performance, game performance, or both. We found that ADHD symptoms were significantly related to RTV for the traditional CPT [r_s_(44) = 0.55, *p* < 0.001], and were related to RTV for the game CPT only at a “trend” level [r_s_(44) = 0.28, *p* = 0.06]. The magnitude of these correlations was also different from one another (*p* = 0.01), indicating that the relationship between ADHD symptoms and RTV was stronger on the traditional CPT compared the game CPT. This suggests that ADHD symptoms are strongly related to RTV on the traditional CPT and the effect of ADHD on performance was reduced with the addition of game features. Moreover, these results demonstrate that those with more ADHD symptoms had better performance on the game CPT, while those with fewer symptoms had better performance on the traditional CPT.

For the sustained attention condition, ADHD symptoms were not significantly related to the game change score for any of our performance metrics [RT: r_s_(46) = −0.11, *p* = 0.45; RTV: r_s_(46) = −0.07, *p* = 0.62; d-prime: r_s_(46) = −0.02, *p* = 0.92; all *p*_corrected_ = 1.00; Figure 4A, right]. Importantly, the magnitude of these correlations was also different between the inhibitory control and sustained attention conditions for RTV (Z = 2.48, *p* = 0.01) and at a “trend” level for d-prime (Z = 1.90, *p* = 0.06), but not for RT (Z = 1.03, *p* = 0.30). This demonstrates that the ADHD-game change relationship was more strongly pronounced for the CPT condition in which many targets were present (inhibitory control condition), specifically for attention consistency (RTV).

### Reward responsiveness

For the inhibitory control condition, reward responsiveness was positively related to the game change score for RTV [r_s_(44) = 0.38, *p* = 0.009, *p*_corrected_ = 0.05; Figure 4B, left], but not for RT [r_s_(44) = 0.26, *p* = 0.08, *p*_corrected_ = 0.48] or d-prime [r_s_(44) = 0.17, *p* = 0.26, *p*_corrected_ = 1.00; Figure 4B, right]. The direction of these results suggests that the higher the reward responsiveness, the greater the advantage for the game CPT compared to the traditional CPT, specifically in terms of attention consistency (RTV). Similar to ADHD symptoms, it is important to note that this metric (inhibitory control RTV) showed no mean difference between the traditional and game CPTs (Figure 3B), suggesting that reward responsivity indexes variability in performance changes with game features in the absence of overall effects.

As with ADHD symptoms, we next focused on RTV for the inhibitory control condition, given that we found a relationship between reward responsivity and the game change score for this metric. We found that reward responsiveness was numerically more strongly related to RTV for the traditional CPT [r_s_(44) = 0.25, *p* = 0.10] than the game CPT [r_s_(44) = −0.03, *p* = 0.86]. The magnitude of these correlations was different from one another (*p* = 0.02), suggesting that those with more reward responsiveness had better performance on the game CPT while those with less responsiveness had better performance on the traditional CPT. Further, these results demonstrate that the effect of reward responsiveness on performance was reduced with the addition of game features.

For the sustained attention condition, reward responsiveness was not related to the game change score for any of our performance metrics [RT: r_s_(46) = 0.09, *p* = 0.54; RTV: r_s_(46) = 0.16, *p* = 0.29; d-prime: r_s_(46) = 0.18, *p* = 0.23; all *p*_corrected_ = 1.00; Figure 4B, right]. However, the magnitude of these correlations was not different between the inhibitory control and sustained attention conditions for RTV (Z = 1.17, *p* = 0.24), d-prime (Z = −0.05, *p* = 0.96) or for RT (Z = 0.86, *p* = 0.39). Similar to our ADHD symptom findings, this demonstrates that the relationship between reward responsiveness and game change was numerically (but not statistically) more strongly pronounced for the CPT condition in which many targets are present (inhibitory control), most notably for attention consistency (RTV).

### Age

For the inhibitory control condition, age was negatively related to the game change score for both RTV [r_s_(44) = −0.40, *p* = 0.006, *p*_corrected_ = 0.04] and d-prime [r_s_(44) = −0.44 *p* = 0.002, *p*_corrected_ = 0.01], but not for RT [r_s_(44) = 0.02, *p* = 0.88, *p*_corrected_ = 1.00; Figure 4C, right]. This suggests that younger participants performed better on the game CPT compared to the traditional CPT, both in terms of their attention consistency (RTV) and their responding accuracy (d-prime). Similar to our other results, it is important to note that these metrics (inhibitory control RTV and d-prime) showed no mean difference between the traditional and game CPTs (Figure 3B), suggesting that age indexes variability in performance changes with game features in the absence of overall effects.

For the sustained attention condition, age was also negatively related to the game change score for RTV [r_s_(46) = −0.40, *p* = 0.005, *p*_corrected_ = 0.03; Figure 4C, left], but not for RT [r_s_(46) = 0.07, *p* = 0.66, *p*_corrected_ = 1.00] or d-prime [r_s_(46) = −0.32, *p* = 0.03, *p*_corrected_ = 0.18; Figure 4C, right]. Notably, in line with our other results, RTV for the sustained attention condition also did not show a mean difference between the traditional and game CPTs (Figure 3B). Further, the magnitude of these correlations was not different between the inhibitory control and sustained attention conditions for any metric (RT: Z = −0.25, *p* = 0.81; RTV: Z = 0.00, *p* = 1.00; d-prime: Z = −0.69, *p* = 0.49). Unlike our findings for ADHD symptoms and reward responsiveness, this suggests that the age influence on the game change score was present for both CPT conditions, regardless of the number of target stimuli (i.e., frequent or infrequent).

As with ADHD symptoms and reward responsiveness, we next focused on RTV changes due to game features, given that we found a relationship between age and the game change score for this metric. We similarly found that age was related to RTV for the traditional CPT [r_s_(46) = −0.45, *p* = 0.001], but not for the game CPT [r_s_(46) = −0.03, *p* = 0.82]. Further, the magnitude of these correlations was significantly different from one another (*p* < 0.001), providing evidence that younger individuals had better performance on the game CPT in the sustained attention condition while older individuals had better performance on the traditional CPT. Further, these results demonstrate that the effect of age on performance was reduced with added game elements. We also found a similar pattern of results for the inhibitory control condition.

### Video game play

We did not find relationships between frequency of video game play (hours per week) and the game change score for any metrics for the inhibitory control condition [RT: r_s_(44) = −0.31, *p* = 0.04, *p*_corrected_ = 0.24; RTV: r_s_(44) = −0.03, *p* = 0.85, *p*_corrected_ = 1.00; d-prime: r_s_(44) = 0.20, *p* = 0.19, *p*_corrected_ = 1.00]. We found similar results for the sustained attention condition [RT: r_s_(46) = 0.05, *p* = 0.75, *p*_corrected_ = 1.00; RTV: r_s_(46) = 0.02, *p* = 0.89, *p*_corrected_ = 1.00; d-prime: r_s_(46) = 0.27, *p* = 0.07, *p*_corrected_ = 0.42].

### Gender

We did not find gender differences on the game change score for any metrics for the inhibitory control condition [RT: t(44) = 0.22, *p* = 0.83; RTV: t(44) = −0.35, *p* = 0.73; d-prime: t(44) = −0.17, *p* = 0.87; all *p*_corrected_ = 1.00]. We found similar results for the sustained attention condition [t(46) = 0.55, *p* = 0.59; RTV: t(46) = −0.25, p = 0.81; d-prime: t(46) = −1.36, *p* = 0.18; all *p*_corrected_ = 1.00].

### Years of education completed

We did not find relationships between years of education and the game change score for any metrics for the inhibitory control condition [RT: r_s_(44) = 0.06, *p* = 0.72; RTV: r_s_(44) = −0.02, *p* = 0.91; d-prime: r_s_(44) = −0.11, *p* = 0.46; all *p*_corrected_ = 1.00]. We found similar results for the sustained attention condition [RT: r_s_(46) = 0.11, *p* = 0.44; RTV: r_s_(46) = 0.13, *p* = 0.39; d-prime: r_s_(46) = = 0.20, *p* = 0.17; all *p*_corrected_ = 1.00].

## Discussion

The findings presented here demonstrate that game features have a mixed influence on attention abilities: game features were not related to a change in performance for most metrics, while game features were related to a reduction in performance for a few metrics (i.e., RT and d-prime only for the sustained attention CPT condition). Importantly, we also showed that game features change attention performance for adults with specific profiles. Those with more ADHD symptoms, higher reward responsiveness, and younger age performed better on the game CPT, while those with fewer ADHD symptoms, lower reward responsiveness, and older age performed better on the traditional CPT without game elements. Below we discuss the implications and potential mechanisms of how game features affect attention abilities for particular individuals.

### Game features affect attention performance

We first compared traditional and game CPT performance across all individuals and found that performance was highly correlated between the traditional and game CPT, suggesting that high performers on the traditional CPT also performed well on the game CPT. We next compared mean performances between the traditional and game CPT. Interestingly, game features were related to impaired performance for two of six metrics: processing speed (RT) and accuracy (d-prime), only in the sustained attention condition with infrequent target stimuli. However, there were no differences between the traditional and game CPT for all other metrics: attention consistency (RTV) for the sustained attention condition and all metrics for the inhibitory control condition with frequent target stimuli. Although our null results should be interpreted with caution given our sample sizes and the size of effects we could detect, these results mirror previous equivocal findings on whether game features enhance or impair performance (Lumsden et al., 2016; Khaleghi et al., 2021) and provide compelling evidence that such differential effects could be related to the CPT design, such as the number of target stimuli. In the sustained attention condition, participants may have been distracted by some game features that ultimately impaired their performance as in other work (McPherson and Burns, 2007, 2008; Miranda and Palmer, 2014). In the inhibitory control condition, however, these potentially distracting elements may have been countered by the more frequent targets, feedback, and rewards, resulting in no net performance difference between the game and traditional CPT.

### Individual differences predict whether game features impact attention

Given our mixed findings on whether game features affected attention abilities, we next identified factors that may predict whether certain individuals performed better (or worse) on the game CPT compared to the traditional CPT. First, we found that game features were related to better attention performance in adults who reported more ADHD symptoms, even though they had no formal diagnosis, while they were related to worse attention performance for those with fewer symptoms. These results parallel those in children with ADHD, who can sustain their attention when playing video games (Hinshaw, 2017) or CPTs with added game features (Shaw et al., 2016). Collectively, this provides support for the idea that attention is context-dependent in those with ADHD symptoms (Hinshaw, 2017). As these participants were healthy adults, our results further underscore the importance of examining ADHD as a spectrum-related phenomena and studying those with sub-clinical difficulties (Hinshaw, 2017).

Notably, the change in performance on the game CPT due to ADHD symptoms was most pronounced for RTV, suggesting that game features are most effective at reducing attentional lapses and may have fewer effects on improving speed (RT) and accuracy (d-prime). Although comparable work in children with ADHD only examined d-prime related metrics of error responding (Shaw et al., 2016), our results suggest that RTV may be more strongly affected by game features in adults. In addition, the ADHD-related game change was only evident in the condition with many target stimuli (inhibitory control), in alignment with work in children that used a CPT with frequent targets (Shaw et al., 2016) This interestingly suggests that the ADHD-game change relationship may be titrated by the amount of reward stimuli presented. In the inhibitory control condition, there were many more opportunities to receive points and feedback, compared to the sustained attention condition. In both conditions, however, other features such as storyline, competition, and aesthetics were similar. Response-related reward and feedback may be driving factors in producing a game-based advantage for those with more self-reported ADHD symptoms.

Second, we found that game features influenced attention abilities based on individuals’ reward responsivity. Those who reported more reward responsivity performed better on the game CPT, while those with less responsivity performed better on the traditional CPT. Like ADHD symptoms, the game advantage for those with more reward responsivity was most evident for RTV in the inhibitory control condition, further suggesting that reward and feedback may reduce attentional lapses in these individuals. These results are in-line with prior work demonstrating that the BAS reward responsiveness scale indexes the ability to experience pleasure in the anticipation or presence of rewarding stimuli, and is predictive of a higher degree of adaptive (compared to maladaptive) reward-seeking behaviors (Carver and White, 1994; Taubitz et al., 2015). Here, we extend these findings to rewarding effects conferred by the addition of game features to an attention task. Individuals with more reward sensitivity may be more positively influenced by the addition of such game features, allowing them to better employ attention abilities during a prolonged cognitive task.

These findings also align with mechanisms of ADHD-related attention difficulties that point to concurrent dysfunction in domains such as motivation, reinforcement, and reward processing (Beauchaine and McNulty, 2013). Previous work has suggested that these difficulties are related to underlying hypo-dopaminergic functioning (Volkow et al., 2009), which can have downstream effects on attention. In support of this, prior research has shown that both reinforcement and methylphenidate (a pharmacologic treatment for ADHD that increases dopamine) have comparable effects on improving CPT performance (Bubnik et al., 2015). Moreover, video games have been shown to release dopamine (Koepp et al., 1998), pointing to a potential neurobiological mechanism by which game features may improve attention performance.

Finally, we found that changes in attention performance due to added game features was predicted by participant age. Younger individuals performed better on the game CPT, while older adults had better performance on the traditional CPT without game elements. Interestingly, unlike ADHD symptoms and reward responsiveness, this relationship was evident for both CPT conditions, suggesting that the game advantage for younger participants is related to a more general benefit from game features present in both conditions, such as storyline, competition, and aesthetics. Moreover, the pattern of results across individuals may indicate that older adults might generally show reduced performance with game features (i.e., game change scores all below 0) while young adults may have more variable responsivity to game features (i.e., game change scores both above and below 0). This potential finding could be directly interrogated in future work. These general age effects also agree with reports demonstrating that older adults tend to rate video games as less enjoyable (Boot et al., 2013). Our findings further suggest that older adults might not need game elements to motivate performance, although future work should examine other types of game elements that could benefit older individuals (e.g., social interaction). Further, older adults have reduced reward processing, which may be related to reductions in dopaminergic neuromodulation with age (Eppinger et al., 2011, 2012). As such, potential game-based increases in dopamine may not have as strongly impacted attention in older adults. This supports the need for future investigations exploring the relationship between dopaminergic functioning and attention changes from task game elements.

Interestingly, we found that RTV for the inhibitory control condition exhibited the most consistent relationships with individual difference factors that predicted changes in attention performance due to added game features (although null results with other performance metrics should be interpreted with caution given the size of correlation-based effects we could detect with our sample sizes). Importantly, inhibitory control RTV showed no overall (mean) effects with added game features. Rather, we found that individual profiles such as ADHD symptoms, reward responsiveness, and age could predict variability in responsiveness to game elements, where some individuals showed enhanced performance and others showed impaired performance relative to the traditional CPT. RTV is a well-studied indicator of attention-relevant cognitive abilities that indexes the consistency of attentional deployment during extended task performance (MacDonald et al., 2006; Ziegler et al., 2019). Importantly, increased RTV is linked to impairments in attention and, more generally, aspects of cognitive control (West et al., 2002; Stuss et al., 2003; Sonuga-Barke and Castellanos, 2007). For example, both children with ADHD (Castellanos et al., 2006; Karalunas et al., 2014) and older adults with cognitive impairment (Gorus et al., 2008; Tales et al., 2012; Kälin et al., 2014) exhibit increased RTV. In addition, prior work has shown that periods of high RT stability (lower RTV) coincide with fewer responding errors during attention tasks (Esterman et al., 2012). Our findings here suggest that this aspect of attention may not be affected by game features overall, but instead that individuals have varying responsivity to game features in terms of their RTV-based performance. Importantly, our results identify specific profiles of individuals who have improved (or impaired) RTV due to added game features. Future work could also examine how game features affect moment-to-moment fluctuations in attention during task performance on a finer timescale (Esterman et al., 2012).

## Limitations and conclusions

Although these findings expand our understanding of how game features influence attention for specific individuals, there are several limitations that could be addressed in future work. First, we examined only one type of task. Future work should examine whether the effects of game features generalize across other tasks and even cognitive domains. In addition, it will be important to examine how these effects influence repeated testing or cognitive training, where game elements are often included to increase motivation (Anguera and Gazzaley, 2015; Vermeir et al., 2020). Second, we added many game features to the CPT, resulting in an inability to disentangle which elements contributed most to the game-based changes in performance. Although some results suggest the changes were related to feedback and reward, there should be a future focus to understand specific components driving these effects. Relatedly, we only examined one type of game CPT (i.e., a fishing competition), and future work could directly assess how much these game features were found to be engaging through feedback surveys as well as explore other game narratives. Third, it is worth considering potential limitations of the demographic characteristics of the participants in this study. No participants reported a formal ADHD diagnosis, but future work could examine the influence of formal diagnoses and ADHD subtypes, or identify new ADHD sub-groups that have more pronounced context-dependent attention abilities. In addition, we imposed strict MTurk worker quality criteria to ensure sufficient study motivation and adherence, and prior work has indicated that MTurk workers may be even more attentive to study instructions than other types of online participant pools (Hauser and Schwarz, 2016). Nonetheless, studies in the future should consider how overall motivation and demographics of MTurk participants compare to the general population (Burnham et al., 2018) as well as participant pools in other research contexts, such as traditional “in-lab” research. It would be important to examine whether the results described here generalize to other participant groups and research environments. Finally, future work with larger sample sizes should replicate these initial findings and look at informative interactive or additive effects of individual differences (e.g., using a multiple regression or linear mixed effect model approach).

The present findings provide evidence that game features shape attention in adults and, further, identify factors that predict *for whom* these changes are observed. We demonstrate that game features were related to enhanced attention in those with more ADHD symptoms and higher reward responsivity, as well as in younger individuals. Conversely, we show that game features were related to impaired performance for those with fewer ADHD symptoms, lower reward responsivity, and older adults. These results have important implications for the increasing use of cognitive tasks with game elements to assess and improve cognition, and could be extended to the use of game features in interventions to improve health behaviors (Biddiss and Irwin, 2010; Shoemaker et al., 2015; Sardi et al., 2017; Vajawat et al., 2020) or learning (Griffiths, 2008; Adams, 2009). Our findings emphasize that changes in attention abilities due to game elements depend on underlying individual differences and open new avenues for better understanding who benefits most from the incorporation of game features in cognitive tasks.

## Data availability statement

The raw data supporting the conclusions of this article will be made available by the authors, without undue reservation.

## Ethics statement

The studies involving human participants were reviewed and approved by the Institutional Review Board of the University of California, San Francisco. The patients/participants provided their written informed consent to participate in this study.

## Author contributions

CG, JS, RA-S, JA, and AG designed the study. CG, JS, and RA-S performed testing and data collection. CG and JS analyzed the data. CG, JS, JA, and AG wrote the manuscript. RA-S revised the manuscript. All authors contributed to the article and approved the submitted version.

## Funding

This work was supported by a National Science Foundation SBE Postdoctoral Research Fellowship (Grant 1808384 to CG) and generous support from our Neuroscape donors (Neuroscape Network).

## Conflict of interest

The authors declare that the research was conducted in the absence of any commercial or financial relationships that could be construed as a potential conflict of interest.

## Publisher’s note

All claims expressed in this article are solely those of the authors and do not necessarily represent those of their affiliated organizations, or those of the publisher, the editors and the reviewers. Any product that may be evaluated in this article, or claim that may be made by its manufacturer, is not guaranteed or endorsed by the publisher.
