# Supplementary material for: Influence of game features on attention in adults
Source: Front Psychol. 2023 May 9;14:1123306. doi: 10.3389/fpsyg.2023.1123306 (PMC10203248; doi:10.3389/fpsyg.2023.1123306)
Supplement: Supplementary file 1 [file Table_1.DOCX]

**Influence of game features on attention in adults**

**Supplementary Materials**

Courtney L. Gallen^1,2,†,^**^*^**, Jessica N. Schachtner^1,2,3,†^, Roger Anguera-Singla^1,2^, Joaquin A. Anguera^1,2,4^, Adam Gazzaley^1,2,4,5,^**^*^**

**Author Affiliations:**

^1^Department of Neurology, University of California, San Francisco, USA

^2^Neuroscape Center, University of California, San Francisco, USA

^3^Now at Department of Psychology, University of Arizona, Tucson, AZ, USA

^4^Department of Psychiatry, University of California, San Francisco, USA

^5^Department of Physiology, University of California, San Francisco, USA

^†^These authors share first authorship

***Corresponding authors:**

Adam Gazzaley, [adam.gazzaley@ucsf.edu](mailto:adam.gazzaley@ucsf.edu)

Courtney L. Gallen, [courtney.gallen@ucsf.edu](mailto:courtney.gallen@ucsf.edu)

Sandler Neurosciences Center

675 Nelson Rising Lane, Room 505

San Francisco, CA 94158

(415) 502-7322

**Table S1.** Descriptive statistics for CPT performance

|  | **Inhibitory Control** | | | **Sustained Attention** | | |
| --- | --- | --- | --- | --- | --- | --- |
|  | **RT**  Mean (SD) | **RTV**  Mean (SD) | **D-Prime**  Mean (SD) | **RT**  Mean (SD) | **RTV**  Mean (SD) | **D-Prime**  Mean (SD) |
| Traditional | 0.43 (0.18) | 0.12 (0.10) | 3.69 (1.98) | 0.49 (0.19) | 0.11 (0.07) | 5.88 (2.31) |
| Game | 0.43 (0.20) | 0.12 (0.08) | 3.52 (1.64) | 0.53 (0.24) | 0.12 (0.07) | 4.81 (1.87) |

**Table S2.** Effects of predictor variables on game change scores

|  | **Inhibitory Control** | | | **Sustained Attention** | | |
| --- | --- | --- | --- | --- | --- | --- |
|  | **Δ RT** | **Δ RTV** | **Δ D-Prime** | **Δ RT** | **Δ RTV** | **Δ D-Prime** |
| ADHD | R_s_ = 0.10, p = 0.49 | R_s_ = 0.41, p = 0.004 | R_s_ = 0.35, p = 0.02 | R_s_ = -0.11, p = 0.45 | R_s_ = -0.07, p = 0.62 | R_s_ = -0.02, p = 0.92 |
| Reward | R_s_ = 0.26, p = 0.08 | R_s_ = 0.38, p = 0.009 | R_s_ = 0.17, p = 0.26 | R_s_ = 0.09, p = 0.54 | R_s_ = 0.16, p = 0.29 | R_s_ = 0.18, p = 0.23 |
| Age | R_s_ = 0.02, p = 0.88 | R_s_ = -0.40, p = 0.006 | R_s_ = -0.44, p = 0.002 | R_s_ = 0.07, p = 0.66 | R_s_ = -0.40, p = 0.005 | R_s_ = -0.32, p = 0.03 |
| Video game | R_s_ = -0.31, p = 0.04 | R_s_ = -0.03, p = 0.85 | R_s_ = 0.20, p = 0.19 | R_s_ = 0.05, p = 0.75 | R_s_ = 0.02, p = 0.89 | R_s_ = 0.27, p = 0.07 |
| Education | R_s_ = 0.06, p = 0.72 | R_s_ = -0.02, p = 0.91 | R_s_ = -0.11, p = 0.46 | R_s_ = 0.11, p = 0.44 | R_s_ = 0.13, p = 0.39 | R_s_ = 0.20, p = 0.17 |
| Gender | t = 0.22,  p = 0.83 | t = -0.35,  p = 0.73 | t = -0.17,  p = 0.87 | t = 0.55,  p = 0.59 | t = -0.25,  p = 0.81 | t = -1.36,  p = 0.18 |

*P-values are uncorrected p-values.*

# **Figure S1.** Relationship between age and the game change score for RTV in the inhibitory control condition


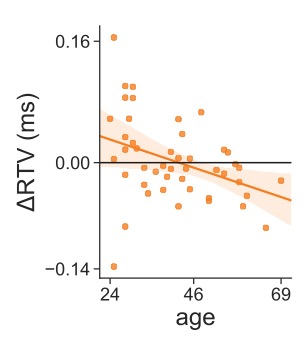


*The line represents the linear fit between the two variables (for visual purposes only). Shaded areas in the scatter plots represent 95% bootstrapped confidence intervals.*

# **Figure S2.** Relationship between age and the game change score for d-prime in the inhibitory control condition


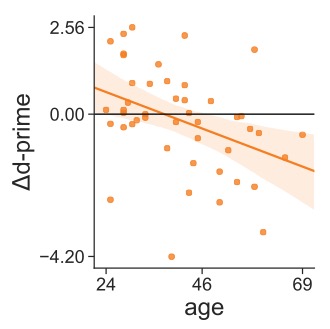


*The line represents the linear fit between the two variables (for visual purposes only). Shaded areas in the scatter plots represent 95% bootstrapped confidence intervals.*
